# Protective Effect of Astragaloside IV on High Glucose-Induced Endothelial Dysfunction via Inhibition of P2X7R Dependent P38 MAPK Signaling Pathway

**DOI:** 10.1155/2020/5070415

**Published:** 2020-09-14

**Authors:** Bin Leng, Cong Li, Yang Sun, Kun Zhao, Ling Zhang, Mei-Li Lu, Hong-Xin Wang

**Affiliations:** ^1^Key Laboratory of Cardiovascular and Cerebrovascular Drug Research of Liaoning Province, Jinzhou Medical University, Jinzhou 121001, China; ^2^First Affiliated Hospital of Jinzhou Medical University, Jinzhou 121001, China

## Abstract

Vascular endothelial dysfunction is associated with increased mortality in patients with diabetes. Astragaloside IV (As-IV) is a bioactive saponin with therapeutic potential as an anti-inflammatory and antiendothelial dysfunction. However, the underlying mechanism for how As-IV ameliorated endothelial dysfunction is still unclear. Therefore, in this study, we examined the protective effect of As-IV against endothelial dysfunction and explored potential molecular biology mechanism. *In vivo*, rats were intraperitoneally injected with streptozotocin (STZ) at a dose of 65 mg/kg body weight to establish a diabetic model. *In vitro* studies, rat aortic endothelial cells (RAOEC) were pretreated with As-IV, SB203580 (p38 MAPK inhibitor) for 2 h prior to the addition of high glucose (33 mM glucose). Our findings indicated that As-IV improved impaired endothelium-dependent relaxation and increased the levels of endothelial NO synthase (eNOS) and nitric oxide (NO) both *in vivo* and *in vitro*. Besides, As-IV treatment inhibited the elevated inflammation and oxidative stress in diabetic model both *in vivo* and *in vitro*. Moreover, As-IV administration reversed the upregulated expression of P2X7R and p-p38 MAPK *in vivo* and *in vitro*. Additionally, the effects of both P2X7R siRNA and SB203580 on endothelial cells were similar to As-IV. Collectively, our study demonstrated that As-IV rescued endothelial dysfunction induced by high glucose via inhibition of P2X7R dependent p38 MAPK signaling pathway. This provides a theoretical basis for the further study of the vascular endothelial protective effects of As-IV.

## 1. Introduction

Diabetes is a chronic metabolic disease characterized by hyperglycemia and widely disturbs the normal metabolic activity of microvascular and macrovascular. Under the influence of long-term hyperglycemia, microangiopathy and macroangiopathy increase the morbidity and mortality of diabetic patients [1]. Endothelial dysfunction, which is manifested by the impairment of nitric oxide (NO)-mediated endothelium-dependent, has been recognized as a common and essential factor in the pathogenesis of diabetic microvascular and macrovascular diseases [2, 3]. In the macrovascular and microvascular endothelium, NO is mainly produced by endothelial NO synthase (eNOS) [2, 4, 5]. Inflammation and oxidative stress are the major triggers for cardiovascular disease and are also the main culprits leading to endothelial dysfunction. The continued existence of both inflammation and oxidative stress exacerbates the reduction of NO bioavailability [6, 7]. Endothelial dysfunction is a complex pathological process that is regulated by multiple regulators, including the activation of P38 mitogen-activated protein kinase (p38 MAPK), which accelerates the occurrence of inflammation and oxidative stress [8, 9]. P2X7 receptor (P2X7R) is an ATP-gated cation channel expressed on the plasma membrane, which is linked to a variety of cardiovascular diseases such as atherosclerosis, thrombosis, and diabetic retinopathy [10]. Although some previous studies indicated that P2X7R is involved in the regulation of inflammation and oxidative stress [11, 12], whether P2X7R is involved in the regulation of diabetes-induced endothelial dysfunction has not been reported yet.

Astragaloside IV (As-IV) is a natural nonsynthetic bioactive saponin extracted from the dried root of Astragalus membranaceus [13] and was widely observed to exert anti-inflammatory and antioxidation effects in various cells and animal models [14–17]. The potent ability of As-IV to restore endothelial dysfunction has also been demonstrated [18] which is consistent with our previous reports [19]. Although there is evidence that As-IV has the potential to prevent endothelial dysfunction, the exact mechanism by which As-IV acts against endothelial dysfunction remains unclear. Hence, this study aimed to further examine whether As-IV could prevent endothelial dysfunction induced by high glucose via inhibition of the P2X7R regulated P38 MAPK signaling pathway.

## 2. Materials and Methods

### 2.1. Animal Experiments

All animal procedures were performed under the principles approved by the Animal Ethics Committee of Jinzhou Medical University. Briefly, Sprague Dawley (SD) rats (obtained from the Experiment Animal Center, Jinzhou Medical University, Jinzhou, China) weighing 220-250 g are adapted to grow for a week in a controlled environment (free access to food and water, 12 h light/dark cycle, and 25 ± 2°C room temperature). Diabetes model was produced by a single dose (65 mg/kg, intraperitoneal injection) of streptozotocin (STZ; Sigma-Aldrich, Shanghai, China) injection. After 72 hours, rats with blood glucose above 16.7 mmol/L can be considered as successful diabetes model. After that, the rats were randomly divided into four groups (*n* = 8): control group (Con), diabetic group (DM), DM + As-IV 40 mg/kg/d (DM40), and DM + As-IV 80 mg/kg/d (DM80). After eight weeks, corresponding samples (aortas and serum) were harvested and kept for following experiments.

### 2.2. Primary Culture of Rat Aortic Endothelial Cells (RAOEC) and siRNA Knockdown of P2X7R

RAOEC were isolated from the aorta of SD rats and cultured according to the method described previously [20]. RAOEC were grown in an endothelial growth medium (PromoCell, Shanghai, China) supplemented with 20% fetal bovine serum (HyClone, Logan, Utah, USA) and maintained in 37°C incubator supplemented with 5% CO2. Cells used in all experiments were below 10 passages. RAOEC were divided into four groups upon reaching 75% confluence: control group (Con), cells cultured in medium (5.5 mM glucose) for 48 h without As-IV and SB203580 (p38 MAPK specific inhibitor); high glucose group (HG), cells were kept in medium containing 33 mM glucose for 48 h; HG + As-IV group (As-IV), after preincubation with As-IV (100 *μ*M) for 2 h, cells were incubated with HG for a further period of 48 h; HG + SB203580 group (SB), after preincubation with SB203580 (10 *μ*M, Selleck, Houston, USA) for 2 h, cells were incubated with HG for a further period of 48 h.

To further study the mechanism of P2X7R in regulating vascular endothelial dysfunction (VED), we knocked down the expression of P2X7R in RAOEC through siRNA. Briefly, RAOEC were transfected with P2X7R siRNA (si- P2X7R, Santa Cruz, Shanghai, China) or a negative control siRNA for 12 h using Lipofectamine 2000. The transfected cells were then incubated in a high glucose environment for another 48 hours.

### 2.3. Vascular Reactivity

SD rats were anesthetized with 20% urethane (1.25 g/kg body weight) [21], and the thorax was opened. The thoracic aorta was then carefully excised and placed in ice-cold PSS buffer (mM): 130 NaCl; 4.7 KCl; 14.9 NaHCO3; 1.17 MgSO47H2O; 11.1 glucose; 0.026 EDTA; 1.16 CaCl2; and 1.18 KH2PO4. After that, the fatty tissue and connective tissue around the thoracic aorta were carefully removed. Subsequently, the rings (about 2 mm in length) of all groups were placed in the chambers (DMT 620M, Danish Myotechnology, Aarhus, Denmark) containing 5 ml PSS solution and maintained at 37°C incubator with 95% O2 and 5% CO2. Next, the endothelial integrity was examined using the relaxation response to acetylcholine (Ach, 10 *μ*M, Sigma-Aldrich, Missouri, USA) after phenylephrine (PE, 10 *μ*M, Sigma-Aldrich, Missouri, USA) preconstriction. Rings with dilation to acetylcholine ≥70% were regarded as endothelial integrity [22, 23]. Then, cumulative concentration-response curves to Ach (10^−9^ to 10^−5^ M) in PE (1 *μ*M) precontracted rings were determined. To determine the contribution of NO to dilatation, the rings from all groups were incubated with NG-nitro-L-arginine methyl ester (L-NAME, NO synthase inhibitor, 100 *μ*M, Selleck, Houston, USA) for 30 min prior to PE-induced contraction.

To determine the role of As-IV, P38 MAPK, and P2X7R in VED, vascular reactivity was measured as previously reported [24] with slight modifications. Briefly, the rings from the control group were incubated with 100 *μ*M As-IV or 10 *μ*M SB203580 for 36 h in the presence or absence of high glucose. Besides, to determine the effects of P2X7R on VED, arterial segments from normal rats were treated with the scramble siRNA or P2X7R siRNA for 8 h, and then, the rings were incubated with high glucose for 36 h. Following incubation, rings were carefully mounted for isometric tension recording in standard organ chambers as mentioned above.

### 2.4. Western Blot

After the heart tissue was removed from the ultralow temperature refrigerator (-86°C), a 20 mg sample was weighed in a clean Eppendorf tube, followed by homogenization and incubation in 100 *μ*L of RIPA lysis buffer containing 1 mM PMSF. The lysed tissue was centrifuged at 12,000 g for 20 min at 4°C. After centrifugation, the protein concentration of the supernatant was measured by the BCA method. A total of 50 *μ*g of protein extract from aorta were separated through 10% SDS–PAGE at 95 V for 100 min followed by transfer onto PVDF membranes. Each membrane was firstly blocked with 5% BSA for 2 h at room temperature and then incubated with primary antibody against eNOS, P2X7R, p38 MAPK, and *β*-actin (Abclonal, Logan, Utah, USA) at 4°C overnight. After several washes with TBST, the HRP Goat anti-Rabbit IgG secondary antibody was added onto the membranes and incubated for 2 h at room temperature. Finally, the membranes were visualized using an ECL detection kit.

### 2.5. Enzyme-Linked Immunosorbent Assay (ELISA)

ELISA was utilized to analyze the protein level of glutathione peroxidase (GSH-px) in the aorta and cells and tumor necrosis factor alpha (TNF-*α*), interleukin 6 (IL-6), Interleukin 18 (IL-18), and Interleukin 1*β* (IL-1*β*) in serum and culture supernatant (R&D Systems, Minneapolis, USA) according to the manufacturer's instructions.

### 2.6. Determination of Superoxide Dismutase (SOD) and NO

The detection of SOD and NO levels in the aorta and cells was performed by Hydroxylamine method with a total SOD assay kit [25] and Nitrate reductase method with a NO assay kit [26] (Nanjing Jiancheng Biotechnology, Nanjing, China) according to the manufacturer's instructions, respectively.

### 2.7. Measurement of Reactive Oxygen Species (ROS)

ROS level was determined by staining vascular tissues and RAOEC with dihydroethidium (DHE, Beyotime, Shanghai, China). Cryosections of aortas (embedded in O.C.T. medium and 7 *μ*m thick) and cells were stained with 5 *μ*M DHE for 30 min at 37°C in the dark. After that, the sections or cells were washed three times with PBS. Images were acquired using a Leica DMI 3000B fluorescent microscope. The fluorescence intensity of ROS was analyzed using the Image J software.

### 2.8. Immunofluorescence Staining

After anesthetizing the rats, intracardial perfusion was performed with 100 mL of normal saline to rinse the blood out of the circulatory system followed by 4% paraformaldehyde. The vascular was removed and placed in 4% paraformaldehyde at 4°C for 24 h. After embedding in paraffin, the vessels were cut into 5 thick slices. Subsequently, slides were deparaffinized in xylene and rehydrated in graded ethanol. Antigen retrieval was achieved by boiling in 10 mM fresh sodium citrate buffer at 121°C for 4 min. After that, slices were treated with 0.3% H_2_O_2_ for 30 min at room temperature to quench endogenous peroxidase. 5% BSA solution was added afterwards to block nonspecific binding. Next, slices were incubated with anti-eNOS antibody at 4°C overnight. A secondary antibody was administrated the next day for 2 h at room temperature. Nuclei were labeled by incubation with the 4′,6-diamidino-2-phenylindole (DAPI).

### 2.9. Statistical Analysis

All the data are presented as mean ± SEM. Statistical analysis was analyzed by one-way ANOVA followed by Bonferroni post hoc tests. *P* value <0.05 was considered statistically significant. Statistical analysis was performed using the SPSS 23.0 software.

## 3. Results

### 3.1. As-IV Protects against Endothelial Dysfunction in STZ-Induced Diabetic Rats

**Figure 1:**
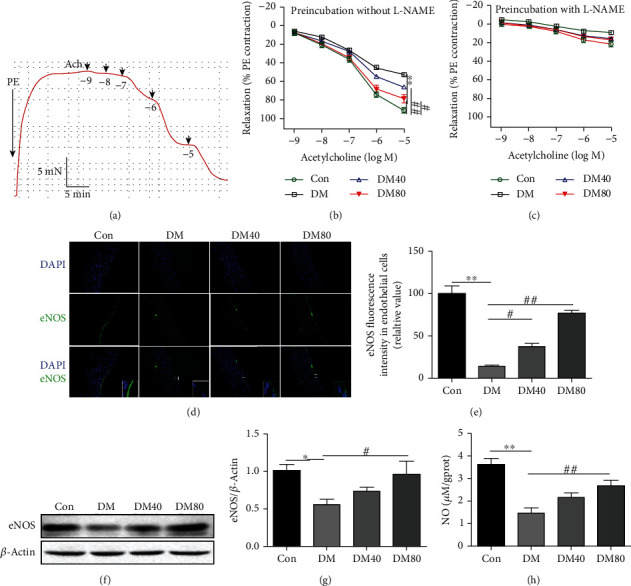
Effect of As-IV on endothelium-dependent relaxation in STZ-induced diabetic rats. (a) Representative typical traces of ACh-stimulated endothelium-dependent relaxation in PE-precontracted aorta. (b, c) Endothelium-dependent relaxation to ACh in the absence or presence of NO synthase inhibitor L-NAME (*n* = 5). (d, e) Representative immunofluorescence staining images and statistical analysis for the protein level of eNOS (*n* = 3), DAPI (blue), and eNOS (green). (f, g) Western blot analysis of the protein level of eNOS with quantification (*n* = 3). (h) The level of NO was tested using the Griess reaction assay (NO reaction end products: nitrate plus nitrite) (*n* = 8). ^∗^*P* < 0.05, ^∗∗^*P* < 0.01 vs. control group. ^#^*P* < 0.05, ^##^*P* < 0.01, vs. DM group.

After diabetic rats were treated with As-IV for 8 weeks, we first investigated the reactivity of aortic rings to assess ACh-mediated endothelium-dependent relaxation in PE-precontracted aortic rings (Figure 1(a)). As depicted in Figure 1(b), As-IV treatment ameliorated the impaired endothelium-dependent relaxation in aortic rings from diabetic rats. To determine the contribution of NO to dilatation, the aortic rings were pretreated with the inhibitor for NO synthase named L-NAME for 30 min. After the rings were preincubated with L-NAME, the ACh-induced relaxation of aortic rings was abolished in all groups (Figure 1(c)). Furthermore, we also used immunofluorescence staining and western blot to determine the protein levels of eNOS in the aorta. Immunofluorescence results showed that As-IV increased high glucose-induced decrease of eNOS in the aortic endothelial cells (Figure 1(d) and 1(e)), while western blot results showed that As-IV reversed the reduction of eNOS expression in the aorta (Figure 1(f)) of STZ-induced diabetic rats. Moreover, the Griess reaction assay showed that the level of NO in the aorta was decreased in DM group, which was reversed by As-IV (Figure 1(h)).

### 3.2. As-IV Administration Prevents STZ-Induced Inflammation and Oxidative Stress in Diabetic Rats

**Figure 2:**
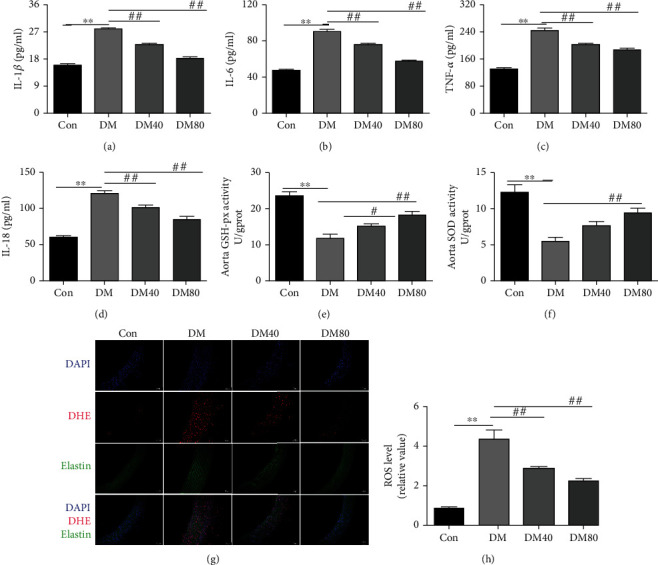
Effect of As-IV on inflammation and oxidative stress in STZ-induced diabetic rats. (a–e) The expression level of IL-6, TNF-*α*, IL-1*β*, and IL-18 proteins and the activity of GSH-px were measured by ELISA (*n* = 8). (f) The level of SOD in the aortas was measured using a SOD assay kit (*n* = 8). (g, h) The level of ROS was determined by DHE staining (*n* = 3). DAPI (blue), DHE dye (red), and elastin autofluorescence signals (green). ^∗∗^*P* < 0.01 vs. control group. ^#^*P* < 0.05, ^##^*P* < 0.01, vs. DM group.

**Figure 3:**
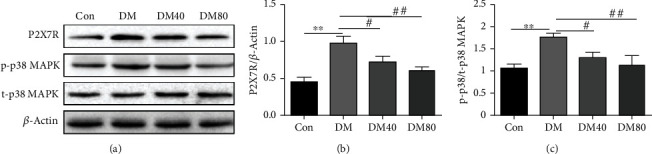
Effect of As-IV on P2X7R, p-p38, and t-p38 MAPK in STZ-induced diabetic rats. (a–c) Western blot analysis of protein levels of P2X7R, p-p38, and t-p38 MAPK with quantification (*n* = 3). ^∗∗^*P* < 0.01 vs. control group. ^#^*P* < 0.05, ^##^*P* < 0.01, vs. DM group.

In order to further investigate the effect of As-IV on endothelial dysfunction in STZ-induced diabetes rats, we measured the levels of inflammation and oxidative stress in the serum and aortas from all groups. As expected, the levels of IL-6, TNF-*α*, IL-1*β*, and IL-18 were enhanced in the serum of STZ-induced diabetic rats, and this was reversed by the treatment of As-IV (Figures 2(a)–2(d)). Moreover, compared with the control group, the levels of SOD and GSH-px were decreased in the aortas from STZ-induced diabetic rats which were reversed by As-IV application (Figures 2(e) and 2(f)). Besides, As-IV mitigated the increased ROS level in the aortas of STZ-induced diabetic rats (Figures 3(g) and 3(h)).

### 3.3. As-IV Treatment Inhibits the STZ-Induced Activation of P2X7R and P38 MAPK Signaling Pathways in Diabetic Rats

Considering the pivotal role of P2X7R in the regulation of P38 MAPK pathway, we sought to determine whether P2X7R regulates endothelial dysfunction through the P38 MAPK signaling pathway in diabetic rats. Our results indicated that the expression level of P2X7R in the aorta of diabetic rats was significantly increased (Figures 3(a) and 3(b)). While the level of P2X7R was dramatically decreased in the aorta of STZ-induced diabetic rats when subjected to 8 weeks of As-IV administration. Besides, the levels of p-P38 MAPK were increased in STZ-induced diabetic rats, which were reversed by As-IV treatment (Figures 3(a) and 3(c)).

### 3.4. As-IV, SB203580, and P2X7R siRNA Treatment Ameliorate Endothelial Dysfunction Induced by High Glucose *In Vitro*

**Figure 4:**
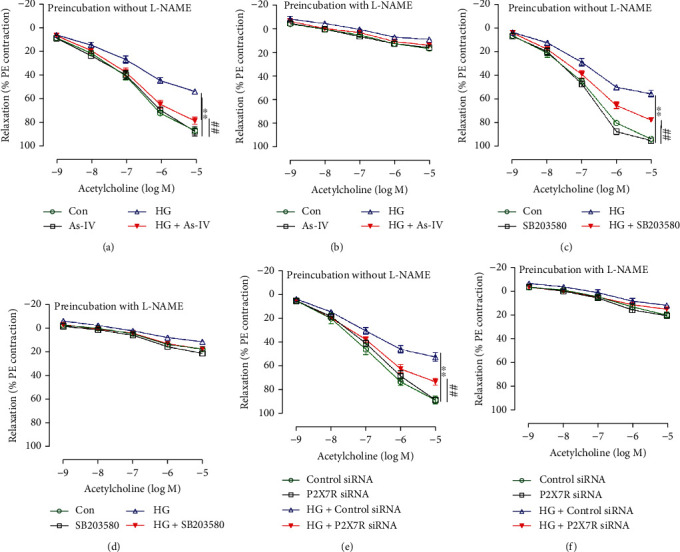
Effect of As-IV, SB203580 and P2X7R siRNA on endothelial dysfunction induced by high glucose (HG) *in vitro*. (a–f) Endothelium-dependent relaxation to ACh in the absence or presence of NO synthase inhibitor L-NAME (*n* = 5). ^∗∗^*P* < 0.01 vs. control group. ^##^*P* < 0.01 vs. HG group.

To determine the role of As-IV and P38 MAPK in high glucose-induced endothelial dysfunction, we pretreated the thoracic aortic rings with As-IV and the p38 MAPK specific inhibitor SB203580 for 48 h in the presence or absence of high glucose (to mimic the chronic hyperglycemia environment of diabetes). The treatment of the thoracic aortic rings with As-IV and SB203580 ameliorated the impaired endothelium-dependent relaxation caused by high glucose and this response was abolished by treatment with L-NAME (Figures 4(a)–4(d)). Moreover, in order to investigate the effects of P2X7R on endothelial dysfunction induced by high glucose, the rings from control rats were incubated with the control siRNA or P2X7R siRNA for 8 h before the rings were incubated with high glucose for another 36 h. Our data indicated that ACh-induced vasodilation was impaired by high glucose treatment, while P2X7R siRNA restored endothelium-dependent vasorelaxation to ACh in high glucose-treated aortic rings (Figures 4(e) and 4(f)).

### 3.5. As-IV and SB203580 Increases eNOS and NO Levels in RAOEC

**Figure 5:**
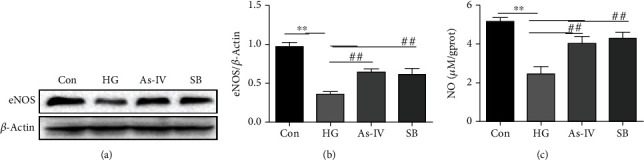
Effect of As-IV and SB203580 on eNOS and NO levels in a high glucose environment. (a, b) Western blot analysis of the protein level of eNOS with quantification (*n* = 3). (c) The level of NO was tested using the Griess reaction (NO reaction end products: nitrate plus nitrite) (*n* = 8). ^∗^*P* < 0.05, ^∗∗^*P* < 0.01, vs. control group. ^##^*P* < 0.01 vs. HG group.

To investigate whether As-IV protects against endothelial dysfunction induced by high glucose through the P38 MAPK signaling pathway, we isolated and cultured endothelial cells from SD rat aorta with or without As-IV and SB203580 in a high glucose environment for 48 h to further examine the effect of As-IV and SB203580 on eNOS and NO levels in RAOEC. Results showed that high glucose decreased the expression level of eNOS and NO which were reversed by As-IV and SB203580 (Figures 5(a)–5(c)).

### 3.6. As-IV and SB203580 Administration Inhibits Inflammation and Oxidative Stress Induced by High Glucose in RAOEC

**Figure 6:**
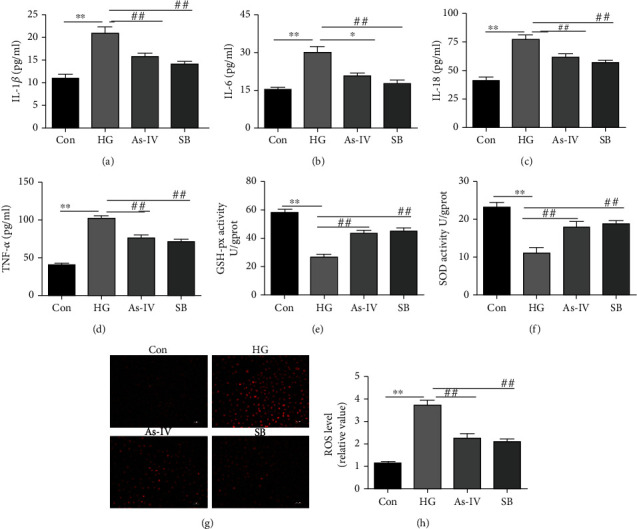
Effect of As-IV and SB203580 on inflammation and oxidative stress induced by high glucose in RAOEC. (a–e) IL-6, TNF-*α*, IL-1*β*, and IL-18 proteins, and GSH-px activity were measured by ELISA (*n* = 8). (f) The level of SOD in RAOEC was measured using a SOD assay kit (*n* = 8). (g, h) The level of ROS was determined by DHE dye (*n* = 3). DHE dye (red). ^∗∗^*P* < 0.01 vs. control group. ^##^*P* < 0.01 vs. HG group.

As aforesaid that inflammation and oxidative stress are critical factors for the progression of endothelial dysfunction, which was regulated by the P38 MAPK pathway. Our data revealed that the content of IL-6, TNF-*α*, IL-1*β*, and IL-18 were increased in the supernatant of RAOEC incubated in a high glucose environment, while both As-IV and SB203580 inhibited the secretion of these inflammatory factors (Figures 6(a)–6(d)). Besides, the GSH-px and SOD activity in RAOEC was observed to be decreased coordinated with upregulated ROS level when incubated in high glucose environment. Nevertheless, both As-IV and SB203580 reversed these effects of high glucose (Figures 6(e)–6(h)).

### 3.7. As-IV and SB203580 Administration Inhibits P2X7R and p-P38 MAPK Expression in RAOEC

**Figure 7:**
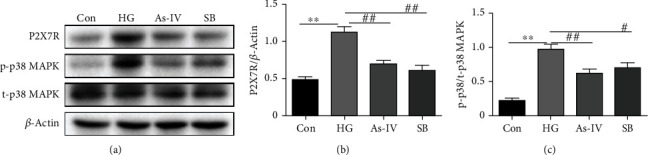
Effect of As-IV and SB203580 administration on P2X7R, p-p38, and t-p38 MAPK in RAOEC. (a–c) Western blot analysis of protein levels of P2X7R, p-p38, and t-p38 MAPK with quantification (*n* = 3). ^∗∗^*P* < 0.01 vs. control group. ^#^*P* < 0.05, ^##^*P* < 0.01, vs. HG group.

To investigate the effect of As-IV on P2X7R and P38 MAPK in RAOEC, we examined the effect of As-IV and SB203580 on P2X7R and P38 MAPK protein expression levels by western blot analysis. After RAOEC was incubated with high glucose, the level of P2X7R and p-P38 MAPK was increased in RAOEC, and this was reversed by treatment with As-IV (Figures 7(a)–7(c)).

### 3.8. P2X7R Knockdown Attenuates Endothelial Dysfunction Induced by High Glucose in RAOEC via P38 MAPK Signaling Pathways

**Figure 8:**
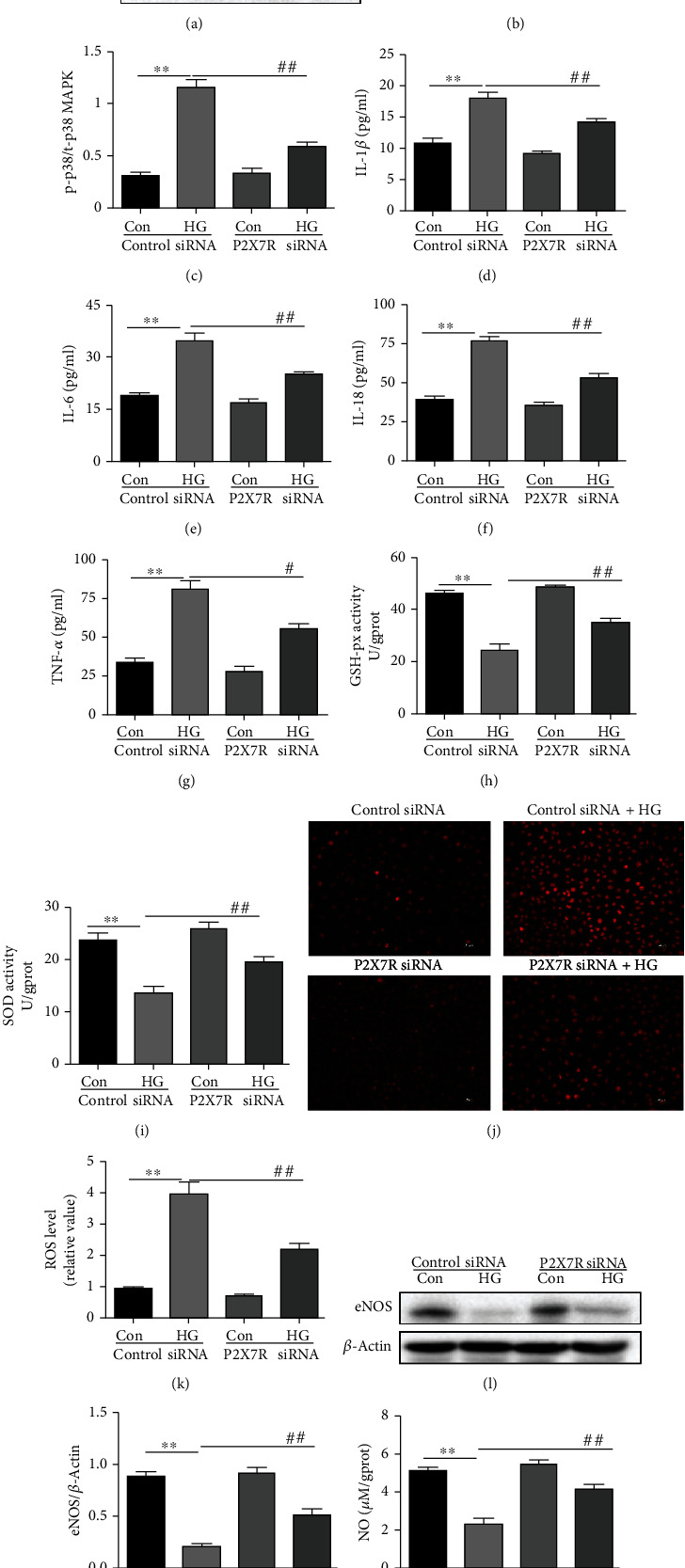
Effect of P2X7R knockdown on P2X7R, p-P38 MAPK, inflammation, oxidative stress, eNOS, and NO in a high glucose environment. (a–c) Western blot analysis of protein levels of P2X7R, p-p38, and t-p38 MAPK with quantification (*n* = 3). (d–h) IL-6, TNF-*α*, IL-1*β*, and IL-18 proteins, and GSH-px activity were measured by ELISA (*n* = 8). (i) The level of SOD in RAOEC was measured using a SOD assay kit (*n* = 8). (j, k) The level of ROS was determined by DHE dye (*n* = 3). DHE dye (red). (l, m) Western blot analyses of the protein level of eNOS with quantification (*n* = 3). (n) The level of NO was tested using the Griess reaction (NO reaction end products: nitrate plus nitrite) (*n* = 8). ^∗∗^*P* < 0.01 vs. control group. ^#^*P* < 0.05, ^##^*P* < 0.01, vs. HG group.

To determine whether P2X7R could be involved in endothelial dysfunction induced by high glucose, RAOEC were transfected with P2X7R siRNA or a control siRNA for 12 h before high glucose incubation for an additional 48 hours. The expression of P2X7R protein in control siRNA group was upregulated after the incubation for in media containing 33 mM glucose. However, pretreatment of P2X7R siRNA was found to downregulate high glucose-stimulated P2X7R expression in RAOEC (Figures 8(a) and 8(b)). In addition, we also observed that high glucose-stimulated upregulation of p-P38 MAPK protein levels was reduced by P2X7R siRNA (Figures 8(a) and 8(c)). Moreover, high glucose increased the secretion of inflammatory cytokines including IL-6, TNF-*α*, IL-1*β*, and IL-18 into the supernatant of RAOEC in the control siRNA group. Furthermore, high glucose treatment reduced the SOD and GSH-px activity and increased the content of ROS, yet all these effects were reversed by P2X7R siRNA (Figures 8(d)–8(k)). More importantly, P2X7R siRNA treatment increased the levels of eNOS and NO in a high glucose environment compared to the control siRNA group (Figures 8(l)–8(n)).

## 4. Discussion

The impaired NO bioavailability due to decreased vascular NO production is widely recognized as the central mechanism responsible for endothelial dysfunction, which could impair endothelium-dependent relaxation [27]. NO is mainly generated by eNOS in the vascular endothelium and then diffuses out of the endothelial cell into subjacent vascular smooth muscle cells to elicit relaxation [28]. After 8 weeks of intraperitoneal injection of STZ in rats, we found that vascular endothelium-dependent relaxation to Ach was diminished, and this change was rescued by As-IV. Additionally, NO production and the eNOS protein level were reduced *in vivo* (STZ induced diabetic rat) and *in vitro* (RAOEC incubated with high glucose), while these effects of high glucose and STZ was reversed by As-IV. Moreover, the protective effect of As-IV on STZ-induced endothelial dysfunction was abolished by the eNOS inhibitor L-NAME, suggesting the most important contribution of NO to these vasomotor responses. These results indicated that As-IV impedes high glucose-induced endothelial dysfunction via activation of the eNOS/NO signaling. Increased oxidative stress and overproduction of inflammatory genes are another hallmarks of endothelial dysfunction [2]. Oxidative stress is caused by the imbalance between oxidizing and antioxidant systems which is manifested by an increase in oxidants (ROS) and a decrease in antioxidants (GSH-px, SOD) [29]. The impairment of endothelium-dependent NO-mediated vasodilation which is mainly due to increased oxidative stress is commonly observed in patients with diabetes [2, 30]. ROS rapidly consumes NO, causing a decrease in NO bioavailability leading to impaired vasorelaxation [2, 6, 27]. Excessive generation of inflammatory cytokines is another major contributing factor to endothelial dysfunction caused by high glucose [31]. Studies have shown that inflammatory factors IL-6, IL-1*β*, IL-18, and TNF-*α* are positively correlated with cardiovascular events and are also involved in the occurrence of endothelial dysfunction [6, 32–35]. Inflammation not only reduces the expression of eNOS but also inhibits the activity of eNOS by preventing the degradation of its endogenous inhibitor asymmetric dimethylarginine (ADMA) [36–38]. ADMA endogenously inhibits eNOS activity which results in decreased vascular NO production and subsequent endothelial dysfunction [37, 39]. Inhibition of excessively activated inflammation and oxidative stress could prevent further deterioration of endothelial dysfunction [36, 40]. Our data suggest that As-IV could inhibit the secretion of inflammatory factors and the activation of oxidative stress.

P38 MAPKs are critical members of the MAPK family and are widely expressed at different levels in almost all human tissues, such as cardiomyocyte and endothelial cells [41, 42]. The P38 MAPK signaling pathway involved in the regulation of inflammation and oxidative stress plays an important role in cardiovascular disease [43, 44]. Besides, P38 MAPK is also involved in the development of endothelial dysfunction [45]. Once activated, P38 MAPK can phosphorylate and promote the recruitment of leukocytes to the site of inflammation [46]. Our results indicate that high-glucose environment enhanced the expression of p-P38 MAPK protein in the aorta and RAOEC, which was reversed by As-IV treatment. To prove that P38 MAPK is involved in the occurrence of endothelial dysfunction, we removed the aorta from healthy rats, and then incubated the aortic rings in a high glucose environment with or without P38 MAPK inhibitor SB203580. The vascular rings were eventually used for isometric tension recording in standard organ chambers. SB203580 treatment ameliorated the impaired endothelium-dependent relaxation in response to Ach in high glucose-treated aortic rings, and L-NAME treatment eliminated this effect. These results suggest that P38 MAPK affects vascular endothelium-dependent relaxation by regulating NO. Furthermore, SB203580 treatment also inhibited the increased inflammation and oxidative stress induced by high glucose. The above results indicate that As-IV inhibits inflammation and oxidative stress through P38 MAPK signaling pathway and ultimately improves endothelial dysfunction induced by high glucose. P2X7R is a ligand-gated nonselective cation channel receptor that is activated by ATP and expressed in endothelial cells [47]. Strong evidence shows that P38 MAPK is regulated by P2X7R [48]. P2X7R aggravates LPS-induced vascular dysfunction and increases downstream production of inflammation [49, 50]. Besides, P2X7R activation leads to endothelial dysfunction by impairing eNOS/NO signaling pathway [51, 52]. However, there is insufficient evidence to suggest that P2X7R is involved in diabetes-induced endothelial dysfunction. In this study, we found that P2X7R expression was increased in the aorta of diabetic rats, while As-IV reduced P2X7R expression. Besides, to study the role of P2X7R in diabetic endothelial dysfunction, we placed the thoracic aortic ring of healthy rats in the medium containing 33 mM glucose in the presence or absence of control siRNA or P2X7R siRNA. The results showed that endothelium-dependent relaxation was impaired by high glucose, while P2X7R knockout reversed this change. Moreover, L-NAME inhibited Ach-caused vasodilatation in all groups, suggesting that P2X7R participates in the regulation of vascular endothelium-dependent relaxation through the NO signaling pathway. In addition, in high glucose-treated RAOEC, P2X7R knockout not only reduced the phosphorylation of P38 MAPK but also inhibited the secretion of inflammatory cytokines and the activation of oxidative stress. More importantly, P2X7R knockout increased eNOS and NO levels. Taken together, our data showed that As-IV improves vascular endothelial dysfunction induced by high glucose via inhibiting P2X7R/P38 MAPK signaling pathway.

**Figure 9:**
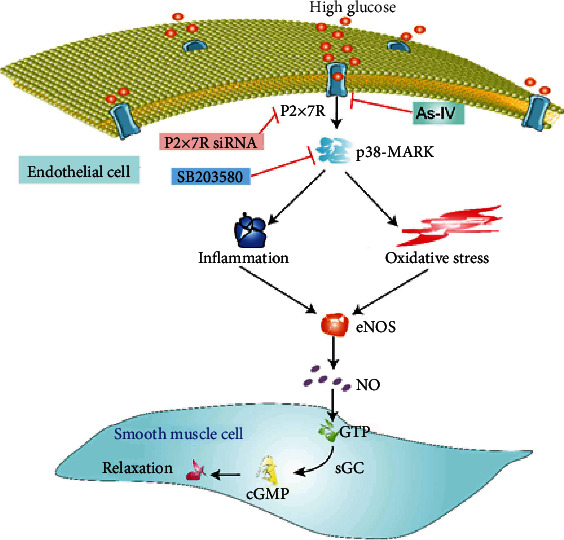
As-IV protects against endothelial dysfunction via inhibition of P2X7R regulated P38 MAPK signaling pathway.

In summary, our data demonstrated for the first time that As-IV reversed the endothelial dysfunction induced by high glucose by inhibiting the activation of P2X7R/P38 MAPK signaling pathway, thereby inhibiting the upregulation of inflammation and oxidative stress, which ultimately improved vascular endothelium-dependent relaxation (Figure 9).

## Data Availability

The data that support the findings of this study are available from the corresponding author, Hongxin Wang, upon reasonable request.
